# Output factors of ionization chambers and solid state detectors for mobile intraoperative radiotherapy (IORT) accelerator electron beams

**DOI:** 10.1002/acm2.12522

**Published:** 2019-01-10

**Authors:** Görkem Güngör, Gökhan Aydın, Teuta Zoto Mustafayev, Enis Özyar

**Affiliations:** ^1^ Department of Medical Physics Medipol University Institute of Health Sciences Istanbul Turkey; ^2^ Department of Radiation Oncology Acıbadem Mehmet Ali Aydınlar University School of Medicine Istanbul Turkey

**Keywords:** intraoperative radiotherapy, LIAC^®^, output factor, recombination factor

## Abstract

**Purpose:**

The electron energy characteristics of mobile intraoperative radiotherapy (IORT) accelerator LIAC ^®^ differ from commonly used linear accelerators, thus some of the frequently used detectors can give less accurate results. The aim of this study is to evaluate the output factors (OFs) of several ionization chambers (IC) and solid state detectors (SS) for electron beam energies generated by LIAC ^®^ and compare with the output factor of Monte Carlo model (MC) in order to determine the adequate detectors for LIAC ^®^.

**Methods:**

The OFs were measured for 6, 8, 10, and 12 MeV electron energies with PTW 23343 Markus, PTW 34045 Advanced Markus, PTW 34001 Roos, IBA PPC05, IBA PPC40, IBA NACP‐02, PTW 31010 Semiflex, PTW 31021 Semiflex 3D, PTW 31014 Pinpoint, PTW 60017 Diode E, PTW 60018 Diode SRS, SNC Diode EDGE, and PTW 60019 micro Diamond detectors. Ion recombination factors (*k*_sat_) of IC were measured for all applicator sizes and OFs were corrected according to *k*_sat_. The measured OFs were compared with Monte Carlo output factors (OF_MC_).

**Results:**

The measured OFs of IBA PPC05, PTW Advanced Markus, PTW Pinpoint, PTW microDiamond, and PTW Diode E detectors are in good agreement with OF_MC_. The maximum deviations of IBA PPC05 OFs to OF_MC_ are −1.6%, +1.5%, +1.5%, and +2.0%; for PTW Advanced Markus +1.0%, +1.5%, +2.0%, and +2.0%; for PTW Pinpoint +2.0%, +1.6%, +4.0%, and +2.0%; for PTW microDiamond −1.6%, +2%, +1.1%, and +1.0%; and for PTW Diode E −+1.7%, +1.7%, +1.3%, and +2.5% for 6, 8, 10, and 12 MeV, respectively. PTW Roos, PTW Markus, IBA PPC40, PTW Semiflex, PTW Semiflex 3D, SNC Diode Edge measured OFs with a maximum deviation of +5.6%, +4.5%, +5.6%, +8.1%, +4.8%, and +9.6% with respect to OF_MC_, while PTW Diode SRS and IBA NACP‐02 were the least accurate (with highest deviations −37.1% and −18.0%, respectively).

**Conclusion:**

The OFs results of solid state detectors PTW microDiamond and PTW Diode E as well as the ICs with small electrode spacing distance such as IBA PPC05, PTW Advanced Markus and PTW Pinpoint are in excellent agreement with OF_MC_. The measurements of the other detectors evaluated in this study are less accurate, thus they should be used with caution. Particularly, PTW Diode SRS and IBA NACP‐02 are not suitable and their use should be avoided in relative dosimetry measurements under high dose per pulsed (DPP) electron beams.

## 1 INTRODUCTION

Intraoperative radiotherapy (IORT) is a treatment technique performed in a suitable operating theater with prescribed dose to the removed tumor bed in a single session during surgery, with the advantage of sparing the critical structures adjacent to irradiation field.^1^, ^2^, ^3^ IORT can be performed with electron beams generated from mobile hard docking or soft docking systems, internal generated kV X‐ray system or remote after loading brachytherapy.^4^, ^5^

LIAC^®^ (SIT S.p.A., Vicenza, Italy) is a light mobile hard docking electron accelerator device designed and dedicated for IORT with a set of flat, 15°, 30°_,_ and 45° bevel angled cylindrical polymethylmethacrylate (PMMA) applicators with various diameters from 30 to 100 mm.^1^, ^6^ It has 10 and 12 MeV models. 12 MeV model produces dose per pulse (DPP) with pulse repetition frequencies (PRF) of 20, 15, 10, and 10 Hz for 6, 8, 10, and 12 MeV electron energies, respectively. Although, LIAC^®^ and conventional linear accelerator (LINAC) can produce electron beams with same energies, their beam characteristics such as energy spectrum, angular distribution, DPP and PRF are quite different.^1^, ^18^ LIAC^®^ has considerably low PRF varying from 1 to 60 Hz in contrast the conventional LINACs work between 200 and 400 Hz.^1^, ^6^, ^7^ Thus, LIAC^®^ can generate DPP of 0.1 to 5 cGy/p which are notably higher than conventional LINACs (around 0.05–0.6 cGy/p).^8^, ^9^

Ionization chambers (IC) are the most frequently used equipment in dosimetry of radiation therapy. Recommended correction factors for IC in IAEA TRS‐381, IAEA TRS‐398, and AAPM TG‐51 dosimetry protocols are determined for conventional LINAC electron beams but not for IORT electron accelerators.^10^, ^11^, ^12^ Because of high DPP, Boag's two voltage analysis (TVA) over estimates the ion recombination factor (*k*_sat_) unfavorably for IC's during direct use for calibration or OFs measurements.^13^, ^14^ In order to overcome inaccuracy with IC measurements of IORT fields, several different correction methods have been proposed in recent years.^13^, ^14^, ^15^, ^16^ Hence it may be more convenient to use less angular, energy and correction factor dependent detectors such as p type diode, natural, or synthetic diamond, Fricke gel dosimetry, electron paramagnetic resonance with Alanine and ionization chambers with small electrode spacing gap. Many studies aimed to determine of the most adequate detectors to use under high DPP electron beams by comparing their OF, however different types and number of detectors compared in each study was limited.^13^, ^14^, ^15^, ^16^, ^17^, ^18^, ^19^, ^20^, ^21^

The aim of this study is to evaluate the OFs and *k*_sat_ responses of several ionization chambers, OFs of solid state detectors for LIAC^®^ electron beam energies and compare the OFs results with MC model in order to determine the suitable detectors for OFs measurements under high DPP (>1 cGy/p) conditions.

## 2 MATERIALS AND METHODS

The OFs of electron beam energies generated by LIAC^®^ for flat applicators were measured with 13 different types of detectors. Plane parallel ion chambers (PPC) were; PTW 23343 Markus, PTW 34045 Advanced Markus, PTW 34001 Roos, IBA PPC05, IBA PPC40, and IBA NACP‐02. Cylindrical ion chambers (CC) were; PTW 31010 Semiflex, PTW 31021 Semiflex 3D, and PTW 31014 Pinpoint. SS detectors were; PTW 60017 Diode E, PTW 60018 Diode SRS, SNC Diode EDGE, and PTW 60019 microDiamond detectors. The detector characteristics are shown in Table 1.

**Table 1:** Detectors and their characteristics

Type of detector	Markus PPC	Advanced Markus PPC	Roos PPC	PPC05	PPC40	PPC NACP‐02
Brand and model	PTW 23343	PTW 34045	PTW 34001	IBA PPC05	IBA—PPC40	IBA NACP‐02
Measurement volume (cc)	0.055	0.02	0.35	0.05	0.40	0.16
Window area density (mg/cm^2^)	106	106	132	176	118	104
Collecting electrode diameter (mm)	5.3	5.0	15.6	10	16	10
Electrode spacing (mm)	2	1	2	0.5	2	2

Type of detector	Semiflex CC	Semiflex 3D CC	Pinpoint CC	Si—Diode E	Si—Diode SRS	microDiamond	Si—Diode
Brand and model	PTW 31010	PTW 31021	PTW 31014	PTW 60017	PTW 60018	PTW 60019	SNC EDGE
Measurement volume (cc)	0.125	0.07	0.015				
Window area density (mg/cm^2^)	78	84	85				
Collecting electrode diameter (mm)	1.1	0.8	0.3				
Sensitive area (mm^2^)				1	1	3.80	0.64
Thickness of volume (μm)				30	250	1	30
Sensitive volume (mm^3^)				0.03	0.3	0.004	0.0192
Total window area density (mg/cm^2^)				140	140	101	
Cover material				0.3 mm RW3 0.4 mm epoxy	0.3 mm RW3 0.27 mm epoxy	0.3 mm RW3 0.6 mm epoxy 0.1 mm Al	0.13 mm Brass

IC, ionization chamber; PPC, plane parallel chamber; CC, cylindrical chamber; RW3, Goettingen White Water; Si, Silica; Al, aluminum.

### 2.A LIAC^®^ 12 MeV model properties

LIAC^®^ is a light mobile electron accelerator without bending magnet dedicated for IORT.^6^ Radiofrequency (RF) power is from 1.2 to 3 MW and provides four clinical energies of 6, 8, 10, and 12 MeV.^1^ The distance between scatter foil and end of applicator is 71.3 cm.^22^ It has seven PMMA applicators in various diameters from 30 to 100 mm with 600 mm long and 5 mm thickness. Beam generation module (BGM) characteristics of LIAC^®^ 12 MeV model which was used in this study, are summarized in Table 2.

**Table 2:** Beam Characteristics of LIAC^®^ 12 MeV model with 6, 8, 10, 12 MeV electron energies

BGM parameters	LIAC^®^ with 100 mm applicator			
Energy (MeV)	6	8	10	12
PRF (Hz)	20	15	10	10
@*R*_100_—depth (mm)	10	13	16	17
@*R*_50_—depth (mm)	22.5	30.1	39.8	46.9
Dose rate (cGy/min)	320	600	900	1200
Dose per pulse (cGy/p)	0.27	0.67	1.50	2.00

### 2.B Output factor and *k*_sat_ measurements

Percentage depth dose (PDD) measurements of electron energies for 100 mm reference applicator were carried out by microDiamond field detector in water phantom (PTW MP3, Freiburg, Germany) in order to obtain *R*_100_ (or *d*_max_) and *R*_50_ parameters. The dosimetric parameters of electron energies which were obtained from PDDs are shown in Table 2.

PPC or CC detectors were not preferred to measure PDDs. It has been shown that parallel plate ionization chambers in high DPP electron beams can be misleading for relative dosimetry.^14^ DPP decreases with depth; as a result this makes *k*_sat_ not constant and diminishes with depth as well. However, if *k*_sat_ is considered constant (*k*_sat_ = 1) PDD is significantly overestimated at greater depths by ICs.^14^ Thus, true PDD can only be obtained by correcting every reading by *k*_sat_ parameter at the depth of IC reading or by using a SS detector instead of ICs. In our study, PDD measurements were performed with a water equivalent SS detector (PTW 60019 microDiamond) in order to obtain *R*_100_ (or *d*_max_) and *R*_50_ parameters.

Dose readings for each field of interest were calculated as the average of three consecutive readings after delivering 300 MU. The OF was calculated as the ratio of reading of any applicator to the reading of reference applicator at *R*_100_ depth for each electron energy.

If the detector was a SS type detector, OF was determined as in equation (1).

(1) $$\text{OF}\left(E,A,d_{\max}\right)=\frac{M{\left(E,A,d_{\max}\right)}_{\mathrm{field}}}{M{\left(E,A_{\mathrm{ref}.},d_{\max}\right)}_{\mathrm{ref}}}$$

$M{\left(E,A,d_{\max}\right)}_{\mathrm{field}}$ is the reading of applicator size *A* at *R*_100_ depth for specific energy. $M{\left(E,A_{\mathrm{ref}.},d_{\max}\right)}_{\mathrm{ref}}$ is the reading of reference applicator where 100 mm applicator is *A*_ref_ at *R*_100_ depth for specific energy.

If the detector was a PPC or CC type ionization chamber, the correction factors were taken into account such as temperature pressure correction factor (*k*_TP_), humidity factor (*k*_h_), electrometer calibration factor (*k*_elec_), polarity factor (*k*_pol_), and *k*_sat_ for OFs measurements of different and reference size of applicators. The general form of OFs determination can be formulated as in equation (2).

(2) $$\frac{\text{OF}\left(E,A,d_{\max}\right)={M\left(E,A,d_{\max}\right)}_{\mathrm{field}}*{\left[k_{\mathrm{TP}}*k_{h}*k_{\mathrm{elec}}*k_{\mathrm{pol}}*k_{\mathrm{sat}}\right]}_{{\left(E,A,d_{\max}\right)}_{\mathrm{field}}}}{M{\left(E,A,d_{\max}\right)}_{\mathrm{ref}}*{\left[k_{\mathrm{TP}}*k_{h}*k_{\mathrm{elec}}*k_{\mathrm{pol}}*k_{\mathrm{sat}}\right]}_{{\left(E,A,d_{\max}\right)}_{\mathrm{ref}}}}$$

*k*_TP_, *k*_h_, and *k*_elec_ factors did not depend on applicator size, energy, DPP. They canceled out each other in equation (2).

Conversely, the effect on a chamber reading of opposite polarity must be checked on relative and absolute measurements. For most chamber types the *k*_pol_ is negligible in photon beams, on the other hand for certain PPC types it has been shown that the polarity effect increases with electron energy and DPP.^11^, ^19^, ^32^ However the effect of polarity is significant for absolute dose calibration measurements,^11^, ^19^ but even if *k*_pol_ is dependent on field size or DPP, the effect is negligible in OFs reading measurements of ICs and cancel out each other in equation (2).

On the other hand, *k*_sat_ has an impact on clinic dosimetry and the correction must be applied to the OFs readings under high DPP.^4^, ^6^, ^8^, ^13^, ^14^, ^15^, ^16^, ^19^, ^21^, ^24^, ^27^ As suggested in previous articles, there are two approaches of *k*_sat_ determination for ICs under high DPP electron beams: Di Martino et al. approach^14^ and Laitano et al. approach.^15^ While Di Martino approach requires intercalibration by using DPP independent dosimeter such as chemical Fricke dosimeter or radiochromic films,^16^ Laitano et al. approach^15^ requires on electrode spacing, applied voltage, calculation parameter p, and chamber type information of ICs. It's a consistent variant of TVA Boag model for high DPP electron beams.

The nominal and one third of nominal voltage dose readings of particular IC were measured for each applicator and *k*_sat_ of particular IC was calculated according to Laitano et al. approach formalism for each applicator size and electron energy.^15^ The required information's of ICs were obtained from table 1 and Laitano et al.^15^ for calculation of *k*_sat_.

Thus the *k*_sat_ corrected OF was determined as in equation (3).

(3) $$\text{OF}{\left(E,A,d_{\max}\right)}_{k_{\mathrm{satcorrected}}}=\frac{M{\left(E,A,d_{\max}\right)}_{\mathrm{field}}*k_{\mathrm{sat}}{\left(E,A,d_{\max}\right)}_{\mathrm{field}}}{M{\left(E,A_{\mathrm{ref}.},d_{\max}\right)}_{\mathrm{ref}}*k_{\mathrm{sat}}{\left(E,A_{\mathrm{ref}.},d_{\max}\right)}_{\mathrm{ref}}}$$

$M{\left(E,A,d_{\max}\right)}_{\mathrm{field}}$ and $M{\left(E,A_{\mathrm{ref}.},d_{\max}\right)}_{\mathrm{ref}}$ are the readings for the applicator sizes of *A* and *A*_ref_, respectively at *R*_100_ depth. $k_{\mathrm{sat}}{\left(E,A,d_{\max}\right)}_{\mathrm{field}}$ and $k_{\mathrm{sat}}{\left(E,A_{\mathrm{ref}.},d_{\max}\right)}_{\mathrm{ref}}$ are the recombination correction factors of ionization chambers for applicator size *A* and *A*_ref_ at *R*_100_ depth, respectively.

The Monte Carlo simulation (OF_MC_) results were obtained from SWL‐LiacSimulation^®^ program provided by the manufacturer. The system runs Monte Carlo simulation based on BEAMnrc/OMEGA and DOSRZnrc.^23^ SWL‐LiacSimulation^®^ requires LIAC^®^ head dimensions, true PDD measurements of all electron beam energies for 30, 50, 70, and 100 mm applicator sizes.^3^, ^5^, ^6^ OF_MC_ was considered as reference output result and the measured OFs of detectors were compared with OF_MC_ for all electron beam energies and applicator sizes.

The “suitable detector” agreement criteria was defined as the percentage difference between measured OFs with OF_MC_ (Δ%) below 2.5% + 1*σ* which was the total uncertainty percentage of output factor determination of LIAC^®^ beam, specified by Iaccarino et al.^3^

## 3 RESULTS

### 3.A Output factors and *k*_sat_ for 6 MeV

**Figure 1:**
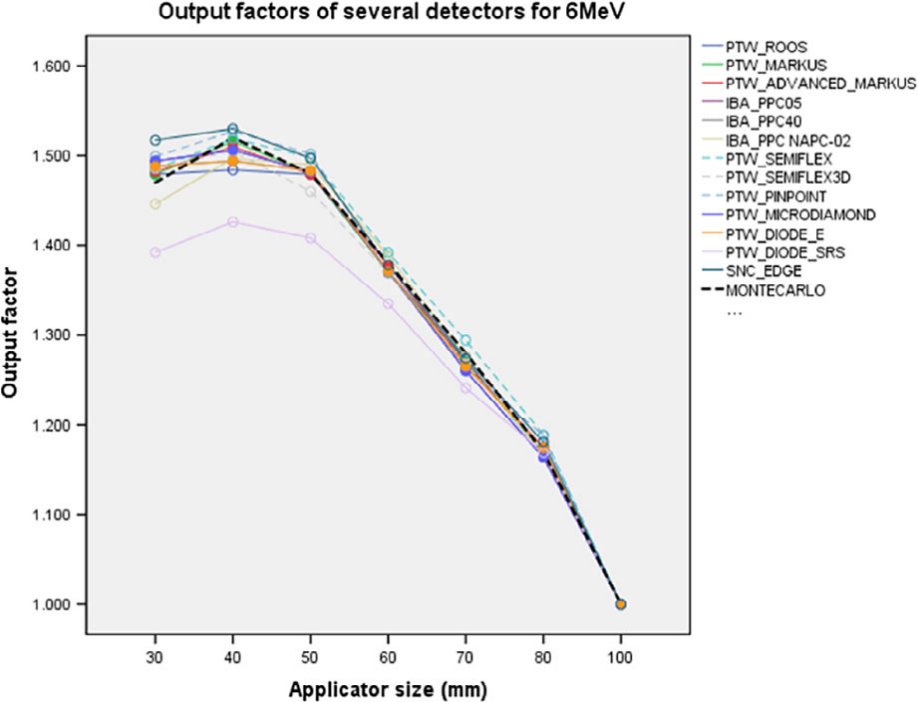
Output factors at 6 MeV for all detectors. Dashed black line is OF_MC_. Dashed lines represent Cylindrical IC detectors and solid lines are PPC and SS detectors. OF_MC_, Monte Carlo output factors; IC, ionization chambers; PPC, plane parallel ion chambers; SS, solid state detectors.

Measured OFs and OF_MC_ for each applicator at 6 MeV are illustrated in Figure 1. Table S1 shows the Δ% difference between OFs and OF_MC_ for all applicators in Supporting information.

The OFs results of PTW Markus, PTW Advanced Markus, IBA PPC05, IBA PPC40, IBA NACP‐02 and PTW Roos PPC chambers, PTW Semiflex, PTW Semiflex 3D and PTW Pinpoint CCs, PTW microDiamond, and PTW Diode E detectors were suitable with respect to OF_MC_. The minimum and maximum Δ% differences were −0.1% and +2%, respectively for all over applicators. However, SNC Edge detector gave the OFs between −0.2% and +3.2%. PTW Diode SRS underestimated the OFs between −6.2% and −0.1% for all applicator sizes.

**Figure 2:**
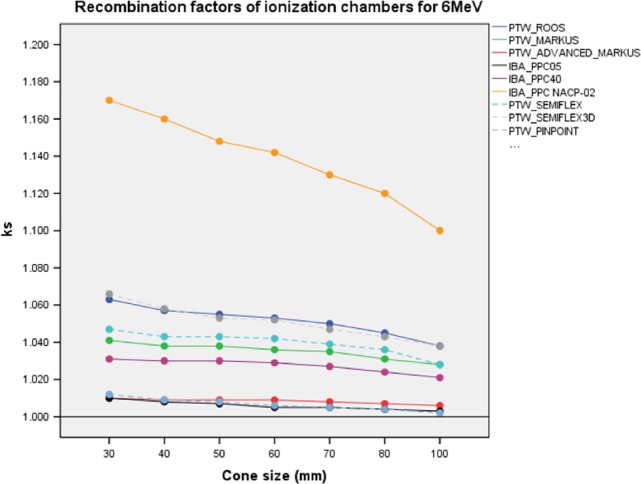
*k*_sat_ of PPC and CC ion chambers with different applicator sizes for 6 MeV. Solid and dashed lines represent PPC and CC ion chambers, respectively. PPC, plane parallel ion chambers; CC, cylindrical ion chambers.

*k*_sat_ of ICs are illustrated in Fig. 2 and represented in Table S2 of Supporting information. The smallest *k*_sat_ ranges were obtained for PTW Pinpoint, PTW Advanced Marcus, and IBA PPC05 ICs. *k*_sat_ ranges of PTW Pinpoint, PTW Advanced Marcus, and IBA PPC05 ICs were [1.010–1.006], [1.012–1.002], and [1.010–1.003] for between 30 and 100 mm applicators, respectively. The maximum *k*_sat_ range was [1.100–1.1700] between 30 and 100 mm applicators for IBA NACP‐02.

### 3.B Output factors and *k*_sat_ for 8 MeV

**Figure 3:**
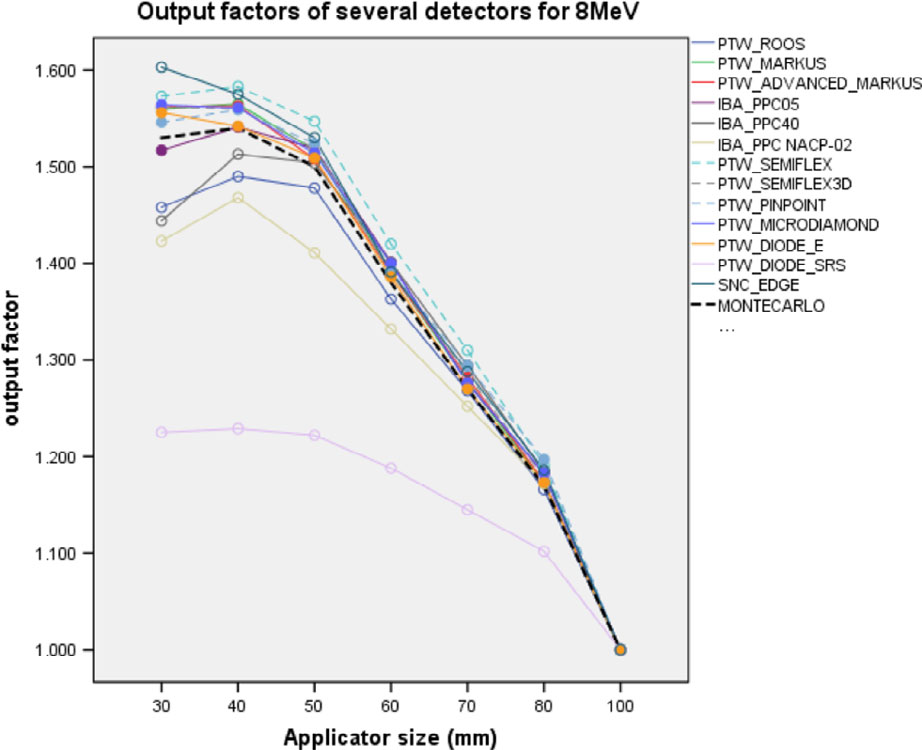
Output factors of 8 MeV for several detectors. Dashed black line is OF_MC_. Dashed lines are Cylindrical IC detectors and solid lines are PPC and SS detectors. OF_MC_, Monte Carlo output factors; IC, ionization chambers; PPC, plane parallel ion chambers; SS, solid state detectors.

Measured OFs and OF_MC_ for 8 MeV are represented in Fig. 3. Table S3 shows the OF values, Δ% difference and OF_MC_ for each applicator size in Supporting information.

PTW Markus, PTW Advanced Markus, IBA PPC05 PPCs, PTW Semiflex 3D, PTW Pinpoint CCs, PTW microDiamond, and PTW Diode E measured the OFs with a minimum and maximum Δ% difference of between +0.0%, and +2.3% with respect to OF_MC_, respectively. These detectors were more suitable than others for this energy. Moreover, the highest Δ% differences for IBA PPC40, IBA NACP‐02 and PTW Roos PPC, PTW Semiflex chambers were −5.6%, −7.0%, −4.7%, and +3.2%, respectively. However, SNC Edge detector overestimated OF +4.8% at 30 mm applicators and conversely, PTW Diode SRS underestimated OFs for all applicators between −20.2% and −5.8%.

**Figure 4:**
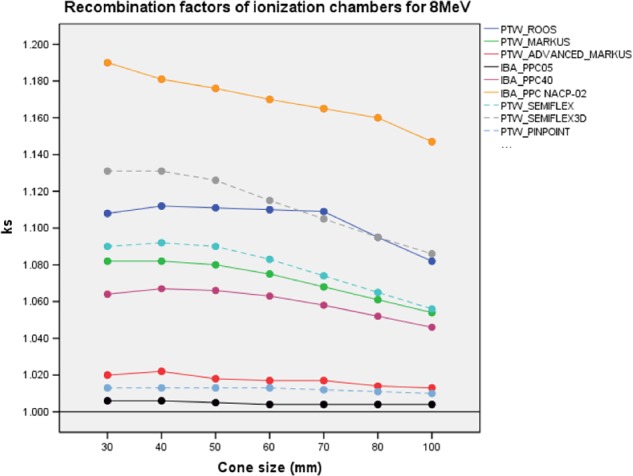
*k*_sat_ of PPC and CC ion chambers with different applicator sizes for 8 MeV. Solid and dashed lines represent PPC and CC ion chambers, respectively. PPC, plane parallel ion chambers; CC, cylindrical ion chambers.

*k*_sat_ ion recombination factors of ICs are illustrated in Fig. 4 and represented in Table S4 of Supporting information. IBA PPC05 was the detector with the least dependence on *k*_sat_, followed by PTW Pinpoint and PTW Advanced Markus IC*. k*_sat_ range was [1.006–1.004] for IBA PPC05; [1.013–1.010] for PTW Pinpoint and [1.022–1.013] for PTW Advanced Markus. The highest *k*_sat_ range was measured by IBA NACP‐02 and varied from [1.147–1.190] for all applicators.

### 3.C Output factors and *k*_sat_ for 10 MeV

**Figure 5:**
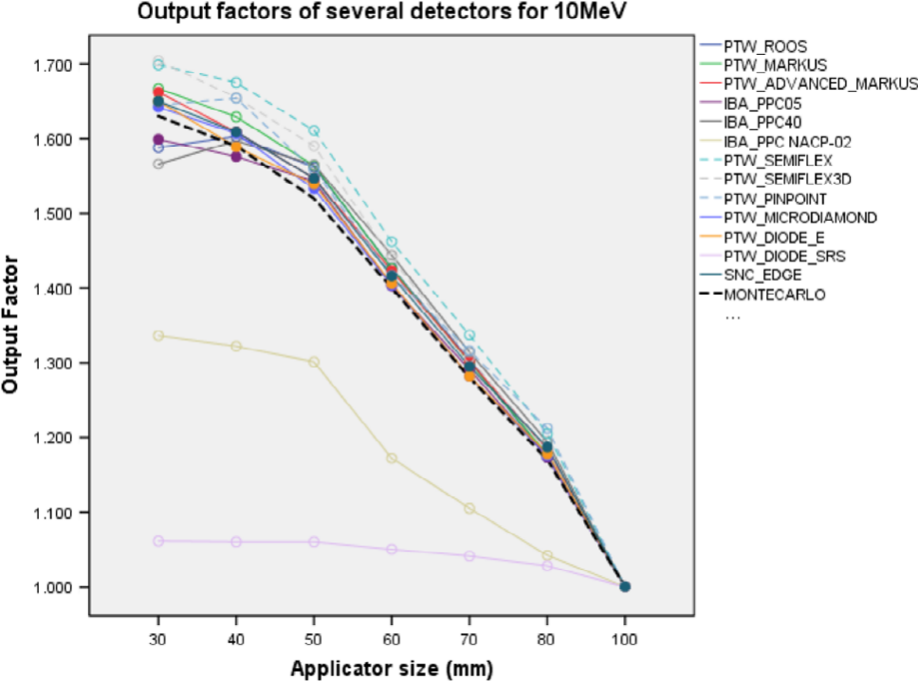
Output factors of 10 MeV for several detectors. Dashed black line is OF_MC_. Dashed lines are Cylindrical IC detectors and solid lines are PPC and SS detectors. OF_MC_, Monte Carlo output factors; IC, ionization chambers; PPC, plane parallel ion chambers; SS, solid state detectors.

Measured OFs, OF_MC_ and Δ% differences for 10 MeV are shown in Fig. 5 and Table S5 in Supporting information. PTW Advanced Markus, IBA PPC05, SNC Edge, PTW microDiamond, and PTW Diode E determined the OFs with a minimum and maximum Δ% difference of between +0.2% and +2.0%, respectively. These detectors were more suitable than others for this energy. The minimum and maximum Δ% differences were +0.6% and 6.0% for PTW Markus, PTW PPC40, PTW Roos and IBA NACP‐02, PTW Semiflex, PTW Semiflex 3D, and PTW Pinpoint CCs, respectively. PTW Diode SRS underestimated OFs between −34.9% and −12.1% with respect to OF_MC_ for all applicators.

**Figure 6:**
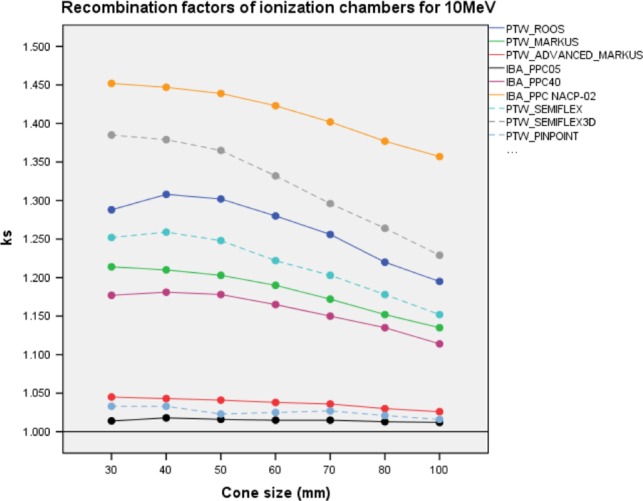
*k*_sat_ of PPC and CC ion chambers with different applicator sizes for 10 MeV. Solid and dashed lines represent as PPC and CC ion chambers, respectively. PPC, plane parallel ion chambers; CC, cylindrical ion chambers.

*k*_sat_ ion recombination factors of ICs are illustrated for 10 MeV energy in Fig. 6 and represented in Table S6 of Supporting information. IBA PPC05, PTW Pinpoint, and PTW Advanced Markus IC were the least dependent detectors to *k*_sat_. The range of *k*_sat_ values was [1.018–1.012] for IBA PPC05; [1.033–1.016] for PTW Pinpoint; and [1.045–1.026] for PTW Advanced Markus. The highest *k*_sat_ range was measured by IBA NACP‐02 and varied from [1.452–1.357] for all applicators.

### 3.D Output factors and *k*_sat_ for 12 MeV

**Figure 7:**
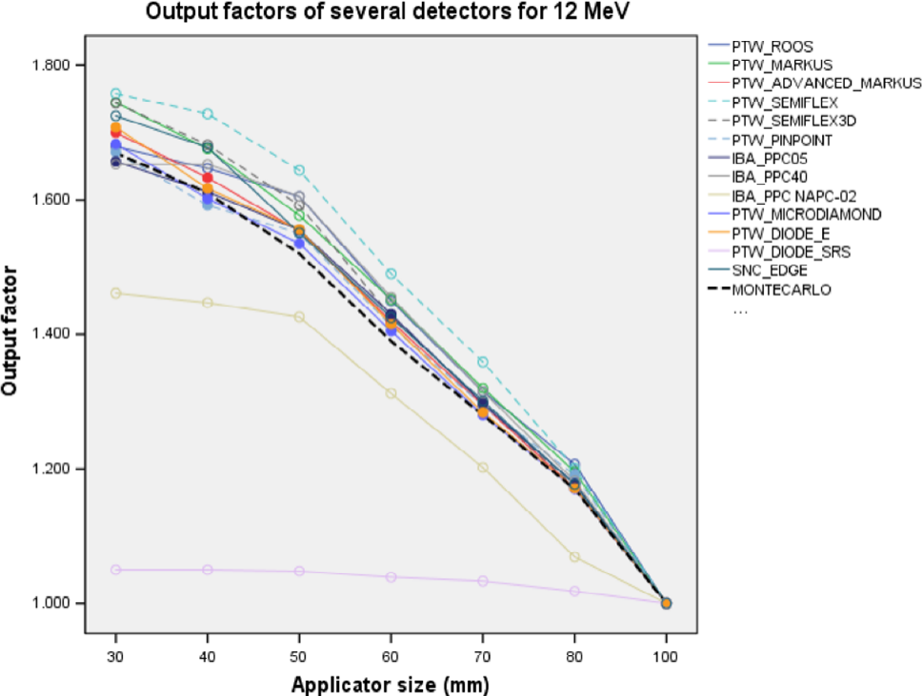
Output factors of 12 MeV for several detectors. Dashed black line is OF_MC_. Dashed lines are Cylindrical IC detectors and solid lines are PPC and SS detectors. OF_MC_, Monte Carlo output factors; IC, ionization chambers; PPC, plane parallel ion chambers; SS, solid state detectors.

**Figure 8:**
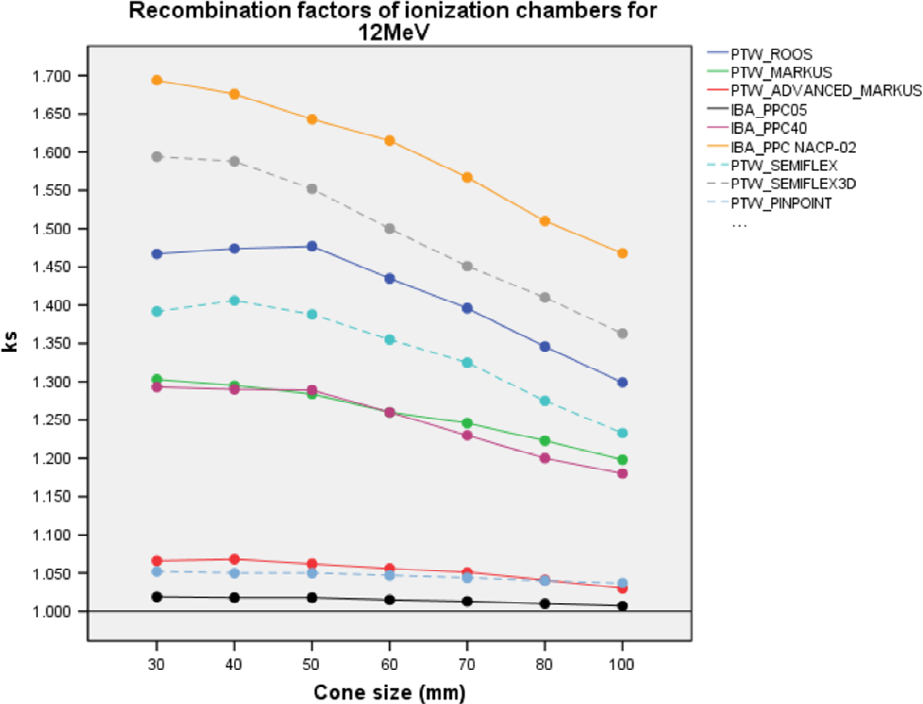
*k*_sat_ of PPC and CC ion chambers with different applicator sizes for 12 MeV. Solid and dashed lines represent as PPC and CC ion chambers, respectively. PPC, plane parallel ion chambers; CC, cylindrical ion chambers.

Measured OF, OF_MC_ and Δ% differences for 12 MeV are illustrated in Figure 7 and shown in Table S7 of Supporting information. *k*_sat_ ion recombination factors are represented in Fig. 8 and Table S8.

PTW Advanced Markus, IBA PPC05, PTW Pinpoint, PTW microDiamond, and PTW Diode E determined the OFs more suitable with +0.1% and +2.4% minimum and maximum differences with respect to OF_MC_, respectively than others. PTW Markus, IBA PPC40, IBA NACP‐02, PTW Roos, PTW Semiflex, PTW Semiflex 3D, and PTW Pinpoint CCs chambers determined OFs with a minimum and maximum deviation of +0.1% and −12.5%, respectively. SNC Edge detector performed over measurement +9.6% at 50 mm and +4.7% at 40 mm applicators. PTW Diode SRS performed lack of measurements and Δ% difference was between −37.1% and −13% with respect to OF_MC_ for all applicators.

IBA PPC05, PTW Pinpoint, and PTW Advanced Markus ICs were the least dependent detectors to *k*_sat_. The range of *k*_sat_ values was [1.019–1.007] for IBA PPC05; [1.052–1.037] for PTW Pinpoint; and [1.066–1.030] for PTW Advanced Markus. The highest *k*_sat_ range was measured by IBA NACP‐02 and varied from [1.694–1.468] for all applicators.

## 4 DISCUSSION

OF is the ratio of detector reading for the specific applicator to the reading of reference applicator. OF, dose rate, and DPP depend on applicator size of LIAC^®^. While applicator size decreases, OF, dose rate, and DPP increase.^5^, ^16^, ^24^ Furthermore, *k*_sat_ is a function of DPP and changes with applicator size for IC detectors.^3^, ^5^, ^14^, ^15^, ^16^, ^24^ Therefore, OF measurements of ICs should be corrected by *k*_sat_. The ion chamber current saturation depends on: initial recombination caused by the recombination of ions along a single particle track, the effect of incomplete charge collection due to diffusion towards the electrode of opposite polarity and the volume recombination caused by diffusion and electrostatic attraction of charge carriers.^25^, ^26^, ^27^, ^28^, ^29^, ^30^, ^31^ The volume dependent parameter (δ) of recombination is correlated with the electrode distance spacing geometry of ion chamber. The electrode plate distance of IBA PPC 05 and PTW Advanced Markus are 0.5 and 1 mm, respectively while PTW Roos, PTW Markus, IBA PPC040, and IBA NACP‐02 electrode distances are 2 mm. Hence, the results in this study show that IBA PPC 05, PTW Advanced Markus PP chambers, and PTW Pinpoint CC are less dependent to *k*_sat_ under high DPP and have less δ. Conversely PTW Semiflex, PTW Semiflex 3D, PTW Roos, PTW Markus, IBA PPC40, and IBA NACP‐02 chambers are more dependent on *k*_sat_. This is due to less electrode spacing which provides higher filled strength for the same polarity chamber voltage.^26^ Furthermore, even if the agreement found significant between OFs and OF_MC_ results of PTW Roos chamber for 30 and 40 mm collimators at 6 and 12 MeV, its certain that when the electron fields are getting smaller, also small field dosimetry and lateral charged particle (LCP) disequilibrium have to be taken in account. This kind of chambers are not suitable for small field measurements because of volume effect of ion chambers.

Notably, IBA PPC05, PTW Advanced Markus, PTW Pinpoint, PTW microDiamond, and PTW Diode E detectors have in superior agreement results. The maximum deviations between OFs and OF_MC_ of IBA PPC05, PTW Advanced Markus, PTW Pinpoint (except 8 MeV), PTW Diode E, and PTW microDiamond are below +2.5%, respectively. Iaccarino et al.^3^ has stated the quadratic dose measurement uncertainties were ±2% + 1*σ* for ICs and ±1.5% + 1*σ* for long term accelerator output fluctuation, thus total OFs determination uncertainty of LIAC^®^ beam was ±2.5% + 1*σ*. Hence these five detectors (PTW microDiamond, PTW Diode E, IBA PPC05, PTW Advanced Markus and PTW Pinpoint) show good agreement with MC results in terms of OFs measurements.

IORT dedicated LIAC^®^ treatments are delivered with single high dose under high DPP (>1 cGy/p) electron energies during surgery.^14^ In this context, electron dosimetry with high DPP requires dosimetry attention when performed by IC. Foremost, the relative and absolute dosimetry characteristics of electron energies produced by LIAC^®^ such as, dose to water (*D*_w_), *k*_sat_, OF, and PDD are affected by DPP. Moreover, International dosimetry protocols suggest TVA method for *k*_sat_ evaluation but this method over estimates and not applicable.^14^, ^15^, ^19^, ^24^ Thus, suitable DPP independent chemical, film dosimeters, solid state detectors with silicon or diamond, and IC with cylindrical or plane parallel detectors are supposed to be investigated in order to measure correct relative and absolute dosimetry for >1 cGy/p electron energies.

In our study, we measured *k*_sat_ and *k*_sat_ corrected OFs with PTW Markus, PTW Advanced Markus, PTW Roos, IBA PPC05, IBA PPC40, IBA NACP‐02 plane parallel chambers, PTW Semiflex, PTW Semiflex 3D, and PTW Pinpoint cylindrical chambers. Also, OFs were measured with PTW Diode E, PTW Diode SRS, PTW microDiamond, and SNC Edge solid state detectors for all electron energies of LIAC^®^ 12 MeV model and showed the Δ% differences with OF_MC_ for flat applicators.

One of the main aims of our study was to investigate the dosimetry characteristics of solid state detectors under DPP electron beam conditions. Both PTW Diode E and PTW Diode SRS are unshielded, p‐type disk shaped, perpendicular to detector axis waterproof silicon diodes. Even though both are p type silicon diode, Diode E demonstrated convenient OF results but conversely Diode SRS measured exceedingly unfavorable worst results with respect to OF_MC_. The reason can be PTW Diode E is designed for both electron and photon radiation qualities while PTW Diode SRS has been designed only for low energy photon detection.

Several authors published the OFs and *k*_sat_ results as a comparison analysis during high DPP irradiation for different types of detectors. Piermattei et al.^19^ used Fricke, MD‐55‐2 radiochromic film, PTW Markus, PTW Roos, and IBA NACP‐02 to measure absorbed dose and *k*_sat_ for NOVAC7 IORT electron accelerator (S.I.T.—Sordina IORT Technologies). They concluded although MD‐55‐2 was independent from high DPP, electron beam calibration was time consuming and unsuitable for IORT. Thus the use of plane parallel ionization chambers was fundamental. But PTW Markus obtained overestimation of *k*_sat_ up to 20% if conventional dosimetric calibration protocols were used.

De Angelis et al.^13^ compared OFs of open and beveled applicators with Alanine and Fricke dosimetry for NOVAC7. They obtained underestimated doses by −2.4% for small open (40 mm) and beveled (22.5° and more) applicators by Fricke dosimeter and Alanine dosimeter gave more accurate beam output determination compared to the Fricke dosimeter.

Björk et al.^17^, ^18^ compared OFs of PTW 60003 natural diamond, IBA Hi‐pSi electron field diode, PTW Advanced Markus, and Monte Carlo for 6, 12, and 20 MeV degraded electron beams generated by Philips/Elekta SL25 LINAC. It was shown that the natural diamond obtained excellent OF results and also diode detector was well suited for electron energies. In concordance with authors’ conclusion, although synthetic microDiamond was used in our study, this type of detector results were in excellent agreement with OF_MC_. The maximum deviations were +1%, +1.1%, +2.0%, and −1.6% for 6, 8, 10, and 12 MeV energies, respectively. Similarly, the maximum deviations were +2.5%, +1.3%, +1.7%, and −1.7% for PTW Diode E. Both PTW microDiamond and PTW Diode E type detectors are suitable for relative dosimetry such as PDD, profile, and OF measurements under higher DPP electron energies.

Di Martino et al.^14^ derived a new ion recombination correction factor formula and absorbed dose measurements were experimentally tested with PTW Roos, PTW Markus chambers for different DPPs which were obtained by Fricke dosimeter for NOVAC7. They found *k*_sat_ increment with DPP and generally it was greater for the Roos than Markus ionization chamber. In this study, the *k*_sat_ parameters increased while applicator sizes diminish and *k*_sat_ values of Roos chamber were greater than Markus chamber. Our results were in agreement with Di Martino et al.^14^

Pimpinella et al.^5^ simulated dosimetric characteristics of electron beams with Monte Carlo and compared the OFs with PTW Markus chamber for NOVAC7. The maximum % differences were obtained −2.5% and −3% at highest energy code D for 6 cm applicator and energy code C for 8 cm applicator. In our study PTW Markus had a maximum % difference of −0.8% (for 30 mm applicator), +1.9% (for 30 mm applicator), +2.9% (for 50 mm applicator), and +4.5% (for 30 mm applicator) compared to OF_MC_ for 6, 8, 10, and 12 MeV energies, respectively. The measurement deviation of PTW Markus chamber increased while electron energy increased, as well.

Cella et al.^16^ compared two different approaches of recombination correction factor calculation under high DPP (Di Martino et al. method^14^ and Laitano et al. method^15^) and their impact on clinical dosimetry by using PTW Markus, PTW Advanced Markus, Fricke II dosimeter for NOVAC7. They used p type Diode to measure PDD and compared the PDD results with *k*_sat_ corrected PDD measurements of PTW Markus and PTW Advanced Markus. They showed that PDD measurements taken with ion chambers should be corrected by *k*_sat_ for every depth to obtain true PDD. These results also mentioned by Di Martino et al. previously.^14^ Laitano et al. approach^15^ depends on a knowledge of chamber characteristics such as electrode spacing, applied voltage, calculation parameter p, and chamber type. It can be described as a variant of Boag model and consistent under high DPP. Conversely, Di Martino et al. method^14^ is independent from Boag model and requires a DPP independent reference dosimeter to obtain *k*_sat_. In our study, although authors concluded Di Martino et al. approach^14^ was safer for proper assessing *k*_sat_, Laitano et al. approach^15^ was used to obtain *k*_sat_ of ICs for every different applicator sizes and electron energies because of absence of DPP independent dosimeter.

Iaccarino et al.^3^ generated Monte Carlo simulation of LIAC^®^ 12 MeV model and compared the OF_MC_ and measured OF with PTW Advanced Markus, IBA PPC05, and PTW Pinpoint IC (for beveled angles). They obtained better than 2% difference between calculated and experimental results for OFs with the exception of smallest applicator which gave difference up to 4% for all energies. Both Pimpinella et al.^5^ and Iaccarino et al.^3^ have shown OFs increase as the applicator size decreases from 100 to 30 mm on NOVAK7 and LIAC^®^ 12 MeV by using ion chambers and MC simulations. The output measurements of our study with PTW Advanced Markus, IBA PPC05, and MC also gave excellent agreement with the authors’ OF results of the same chambers for all energies. However, OF results of 30 mm applicator for 6 MeV gave a maximum 12% difference between this study and Iacarrino et al. results. This unexpected difference could not be explained.

Marrale et al.^9^ compared the OF measurements by means of PTW Markus chamber, Alanine for NOVAC7 model. The obtained results also compared with Geant4 Monte Carlo simulation OF_MC._ They obtained up to 3% difference between PTW Markus OF and OF_MC_ for 10 MeV electron energy of NOVAC7. It was suggested that both Alanine dosimeters and PTW Markus IC might be used if suitable ion recombination factors were used. In our study PTW Markus gave a maximum % difference of −0.8% (at 30 mm applicator), +1.9% (at 30 mm), +2.9% (at 50 mm), and +4.5% (at 30 mm) with compared to OF_MC_ of 6, 8, 10, and 12 MeV energies respectively.

Falco et al.^20^ used PTW microDiamond to measure PDD curves, beam profiles and OFs and compared with those obtained by PTW Advanced Markus ionization chamber for NOVAC11. Although it was concluded that PTW microDiamond was suitable for accurate relative dosimetry, they did not published OF results. Hence we agree with the authors’ conclusion of PTW microDiamond being superior and suitable for relative dosimetry under high DPP conditions but we are unable to compare the results of our study with authors’ OF results.

## 5 CONCLUSION

The OF results of PTW microDiamond and PTW Diode E are in good agreement with OF_MC_. Furthermore, IBA PPC05, PTW Advanced Markus, and PTW Pinpoint are also suitable for OF measurements because they are less dependent to *k*_sat_ than other ICs due to the smaller electrode spacing distance. PTW Roos, PTW Markus, PTW Semiflex, PTW Semiflex 3D, IBA PPC40 detectors are less suitable for OF measurements with respect to smaller electrode spacing chambers and SS detectors. These chambers are more dependent on *k*_sat_ under high DPP and they should be used in relative dosimetry measurements with caution. PTW Diode SRS and IBA NACP‐02 are not suitable and their use should be avoided for relative dosimetry measurements under high DPP electron beams.

## CONFLICTS OF INTEREST

The authors have no relevant conflicts of interest to disclose.

## Supporting information


**Table S1.** Measured and *k*_sat_ corrected OFs for flat applicators at 6 MeV with all detectors and OF_MC_ results. Δ% is the percentage difference of measured OFs to OF_MC._
**Table S2.** *k*_sat_ of PPC and CC ion chambers with different applicator sizes for 6 MeV.
**Table S3.** Measured and *k*_sat_ corrected OFs for flat applicators at 8 MeV of all detectors and OF_MC_ results. Δ% is the percentage difference of measured OFs to OF_MC_.
**Table S4.** *k*_sat_ of PPC and CC ion chambers with different applicator sizes for 8 MeV.
**Table S5.** Measured and *k*_sat_ corrected OFs for flat applicators at 10 MeV of all detectors and OF_MC_ results. Δ% is the percentage difference of measured OFs to OF_MC_.
**Table S6.** *k*_sat_ of ion chambers with different applicator sizes for 10 MeV.
**Table S7.** Measured and k_sat_ corrected OFs for flat applicators at 12 MeV with all detectors and OF_MC_ results. Δ% is the percentage difference of measured OFs to OF_MC_.
**Table S8.** *k*_sat_ of ion chambers with different applicator sizes for 12 MeV.
