# Supplementary material for: Output factors of ionization chambers and solid state detectors for mobile intraoperative radiotherapy (IORT) accelerator electron beams
Source: J Appl Clin Med Phys. 2019 Jan 10;20(2):13–23. doi: 10.1002/acm2.12522 (PMC6370992; doi:10.1002/acm2.12522)
Supplement: Supplementary file 1 — Table S1. Measured and k sat corrected OFs for flat applicators at 6 MeV with all detectors and OFMC results. Δ% is the percentage difference of measured OFs to OFMC. Table S2. k sat of PPC and CC ion chambers with different applicator sizes for 6 MeV. Table S3. Measured and k sat corrected OFs for flat applicators at 8 MeV of all detectors and OFMC results. Δ% is the percentage difference of measured OFs to OFMC. Table S4. k sat of PPC and CC ion chambers with different applicator sizes for 8 MeV. Table S5. Measured and k sat corrected OFs for flat applicators at 10 MeV of all detectors and OFMC results. Δ% is the percentage difference of measured OFs to OFMC. Table S6. k sat of ion chambers with different applicator sizes for 10 MeV. Table S7. Measured and ksat corrected OFs for flat applicators at 12 MeV with all detectors and OFMC results. Δ% is the percentage difference of measured OFs to OFMC. Table S8. k sat of ion chambers with different applicator sizes for 12 MeV. [file ACM2-20-13-s001.docx]

| **Measured OF and OF_MC_ for 6 MeV** | | | | | | | | | | | | | | |
| --- | --- | --- | --- | --- | --- | --- | --- | --- | --- | --- | --- | --- | --- | --- |
| **Applicator Size (mm)** | **Monte Carlo** | **Roos** | **Markus** | **Advanced  Markus** | **PPC 05** | **PPC40** | **PPC NACP-02** | **Semiflex** | **Semiflex 3D** | **Pinpoint** | **microDiamond** | **Diode E** | **Diode SRS** | **Edge** |
| 30 | 1.470 | 1.479 | 1.479 | 1.482 | 1.494 | 1.488 | 1.446 | 1.485 | 1.486 | 1.499 | 1.494 | 1.488 | 1.392 | 1.517 |
| 40 | 1.520 | 1.494 | 1.516 | 1.510 | 1.506 | 1.494 | 1.497 | 1.519 | 1.505 | 1.527 | 1.506 | 1.494 | 1.426 | 1.530 |
| 50 | 1.480 | 1.479 | 1.480 | 1.479 | 1.482 | 1.483 | 1.490 | 1.498 | 1.460 | 1.502 | 1.482 | 1.483 | 1.408 | 1.497 |
| 60 | 1.380 | 1.371 | 1.370 | 1.378 | 1.371 | 1.371 | 1.388 | 1.392 | 1.369 | 1.369 | 1.371 | 1.371 | 1.335 | 1.378 |
| 70 | 1.280 | 1.272 | 1.270 | 1.268 | 1.260 | 1.265 | 1.276 | 1.294 | 1.268 | 1.263 | 1.260 | 1.265 | 1.241 | 1.274 |
| 80 | 1.170 | 1.168 | 1.167 | 1.175 | 1.164 | 1.174 | 1.166 | 1.189 | 1.168 | 1.188 | 1.164 | 1.174 | 1.169 | 1.181 |
| 100 | 1.000 | 1.000 | 1.000 | 1.000 | 1.000 | 1.000 | 1.000 | 1.000 | 1.000 | 1.000 | 1.000 | 1.000 | 1.000 | 1.000 |
| **Measured OF and OF_MC_ Δ% difference for 6 MeV** | | | | | | | | | | | | | | |
| **Applicator Size (mm)** | **Monte Carlo** | **Roos** | **Markus** | **Advanced Markus** | **PPC 05** | **PPC40** | **PPC NACP-02** | **Semiflex** | **Semiflex 3D** | **Pinpoint** | **microDiamond** | **Diode E** | **Diode SRS** | **Edge** |
| 30 | 0.000 | **0.6** | **0.6** | **0.8** | **1.6** | **1.2** | **-1.6** | **1.0** | **1.1** | **2.0** | **1.6** | **1.2** | -5.3 | 3.2 |
| 40 | 0.000 | **-1.4** | **-0.2** | **-0.7** | **-0.9** | **-1.7** | **-1.5** | **-0.1** | **-1.0** | **0.5** | **-0.9** | **-1.7** | -6.2 | **0.7** |
| 50 | 0.000 | **-0.1** | **0.0** | **-0.1** | **0.1** | **0.2** | **0.7** | **1.2** | **-1.3** | **1.5** | **0.1** | **0.2** | -4.9 | **1.1** |
| 60 | 0.000 | **-0.6** | **-0.7** | **-0.2** | **-0.7** | **-0.7** | **0.6** | **0.9** | **-0.8** | **-0.8** | **-0.7** | **-0.7** | -3.2 | **-0.2** |
| 70 | 0.000 | **-0.6** | **-0.8** | **-1.0** | **-1.6** | **-1.2** | **-0.3** | **1.1** | **-0.9** | **-1.3** | **-1.6** | **-1.2** | -3.1 | **-0.5** |
| 80 | 0.000 | **-0.2** | **-0.2** | **0.4** | **-0.5** | **0.3** | **-0.3** | **1.6** | **-0.2** | **1.5** | **-0.5** | **0.4** | **-0.1** | **0.9** |
| 100 | 0.000 | **0.0** | **0.0** | **0.0** | **0.0** | **0.0** | **0.0** | **0.0** | **0.0** | **0.0** | **0.0** | **0.0** | **0.0** | **0.0** |

*Table3. Measured and k_sat_ corrected OFs for flat applicators at 6 MeV with all detectors and OF_MC_ results. Δ% is the percentage difference of measured OFs to OF_MC_*

| *k_sat_* for 6 MeV | | | | | | | | | |
| --- | --- | --- | --- | --- | --- | --- | --- | --- | --- |
| Applicator (mm) | Roos | Markus | Advanced Markus | Semiflex | Semiflex3D | Pinpoint | PPC 05 | PPC40 | PPC NACP-02 |
| **30** | 1.063 | 1.041 | 1.010 | 1.047 | 1.066 | 1.012 | 1.010 | 1.031 | 1.170 |
| **40** | 1.057 | 1.038 | 1.009 | 1.043 | 1.058 | 1.009 | 1.008 | 1.030 | 1.160 |
| **50** | 1.055 | 1.038 | 1.009 | 1.043 | 1.053 | 1.008 | 1.007 | 1.030 | 1.148 |
| **60** | 1.053 | 1.036 | 1.009 | 1.042 | 1.052 | 1.006 | 1.005 | 1.029 | 1.142 |
| **70** | 1.050 | 1.035 | 1.008 | 1.039 | 1.047 | 1.005 | 1.005 | 1.027 | 1.130 |
| **80** | 1.045 | 1.031 | 1.007 | 1.036 | 1.043 | 1.004 | 1.004 | 1.024 | 1.120 |
| **100** | 1.038 | 1.028 | 1.006 | 1.028 | 1.038 | 1.002 | 1.003 | 1.021 | 1.100 |

*Table4. k_sat_ of PPC and CC ion chambers with different applicator sizes for 6 MeV*

| **Measured OF and OF_MC_ for 8 MeV** | | | | | | | | | | | | | | |
| --- | --- | --- | --- | --- | --- | --- | --- | --- | --- | --- | --- | --- | --- | --- |
| **Applicator Size (mm)** | **Monte Carlo** | **Roos** | **Markus** | **Advanced  Markus** | **PPC 05** | **PPC40** | **PPC NACP-02** | **Semiflex** | **Semiflex 3D** | **Pinpoint** | **microDiamond** | **Diode E** | **Diode SRS** | **Edge** |
| 30 | 1.530 | 1.458 | 1.560 | 1.554 | 1.517 | 1.444 | 1.423 | 1.573 | 1.562 | 1.546 | 1.560 | 1.556 | 1.225 | 1.603 |
| 40 | 1.540 | 1.490 | 1.565 | 1.563 | 1.541 | 1.513 | 1.468 | 1.583 | 1.560 | 1.559 | 1.561 | 1.542 | 1.229 | 1.575 |
| 50 | 1.500 | 1.478 | 1.517 | 1.508 | 1.519 | 1.504 | 1.411 | 1.547 | 1.519 | 1.524 | 1.514 | 1.509 | 1.222 | 1.530 |
| 60 | 1.380 | 1.363 | 1.391 | 1.390 | 1.401 | 1.401 | 1.332 | 1.420 | 1.390 | 1.392 | 1.400 | 1.386 | 1.188 | 1.391 |
| 70 | 1.270 | 1.268 | 1.278 | 1.282 | 1.276 | 1.294 | 1.252 | 1.310 | 1.285 | 1.294 | 1.276 | 1.270 | 1.145 | 1.288 |
| 80 | 1.170 | 1.166 | 1.173 | 1.174 | 1.182 | 1.184 | 1.172 | 1.191 | 1.178 | 1.197 | 1.183 | 1.173 | 1.102 | 1.186 |
| 100 | 1.000 | 1.000 | 1.000 | 1.000 | 1.000 | 1.000 | 1.000 | 1.000 | 1.000 | 1.000 | 1.000 | 1.000 | 1.000 | 1.000 |
| **Measured OF and OF_MC_ Δ% difference for 8 MeV** | | | | | | | | | | | | | | |
| **Applicator Size (mm)** | **Monte Carlo** | **Roos** | **Markus** | **Advanced Markus** | **PPC 05** | **PPC40** | **PPC NACP-02** | **Semiflex** | **Semiflex 3D** | **Pinpoint** | **microDiamond** | **Diode E** | **Diode SRS** | **Edge** |
| 30 | **0.0** | -4.7 | **1.9** | **1.5** | **-0.8** | -5.6 | -7.0 | 2.8 | **2.1** | **1.1** | **2.0** | **1.7** | -19.9 | 4.8 |
| 40 | **0.0** | -3.2 | **1.6** | **1.5** | **0.1** | **-1.7** | -4.7 | 2.8 | **1.3** | **1.3** | **1.4** | **0.1** | -20.2 | **2.3** |
| 50 | **0.0** | **-1.5** | **1.2** | **0.5** | **1.2** | **0.3** | -5.9 | 3.2 | **1.3** | **1.6** | **0.9** | **0.6** | -18.6 | **2.0** |
| 60 | **0.0** | **-1.3** | **0.8** | **0.7** | **1.5** | **1.5** | -3.5 | 2.9 | **0.8** | **0.9** | **1.5** | **0.5** | -13.9 | **0.8** |
| 70 | **0.0** | **-0.1** | **0.6** | **0.9** | **0.4** | **1.9** | **-1.4** | 3.1 | **1.2** | **1.9** | **0.5** | **0.0** | -9.8 | **1.4** |
| 80 | **0.0** | **-0.4** | **0.3** | **0.4** | **1.0** | **1.2** | **0.1** | **1.8** | **0.6** | **2.3** | **1.1** | **0.2** | -5.8 | **1.4** |
| 100 | **0.0** | **0.0** | **0.0** | **0.0** | **0.0** | **0.0** | **0.0** | **0.0** | **0.0** | **0.0** | **0.0** | **0.0** | **0.0** | **0.0** |

*Table5. Measured and k_sat_ corrected OFs for flat applicators at 8 MeV of all detectors and OF_MC_ results. Δ% is the percentage difference of measured OFs to OF_MC_*.

| *k_sat_* for 8 MeV | | | | | | | | | |
| --- | --- | --- | --- | --- | --- | --- | --- | --- | --- |
| Applicator (mm) | Roos | Markus | Advanced Markus | Semiflex | Semiflex3D | Pinpoint | PPC 05 | PPC40 | PPC NACP-02 |
| **30** | 1.108 | 1.082 | 1.020 | 1.090 | 1.131 | 1.013 | 1.006 | 1.064 | 1.190 |
| **40** | 1.112 | 1.082 | 1.022 | 1.092 | 1.131 | 1.013 | 1.006 | 1.067 | 1.181 |
| **50** | 1.111 | 1.080 | 1.018 | 1.090 | 1.126 | 1.013 | 1.005 | 1.066 | 1.180 |
| **60** | 1.110 | 1.075 | 1.017 | 1.083 | 1.115 | 1.013 | 1.004 | 1.063 | 1.180 |
| **70** | 1.109 | 1.068 | 1.017 | 1.074 | 1.105 | 1.012 | 1.004 | 1.058 | 1.180 |
| **80** | 1.095 | 1.061 | 1.014 | 1.065 | 1.095 | 1.011 | 1.004 | 1.052 | 1.175 |
| **100** | 1.082 | 1.054 | 1.013 | 1.056 | 1.086 | 1.010 | 1.004 | 1.046 | 1.147 |

*Table6. k_sat_ of PPC and CC ion chambers with different applicator sizes for 8 MeV*

| **Measured OF and OF_MC_ of 10 MeV** | | | | | | | | | | | | | | |
| --- | --- | --- | --- | --- | --- | --- | --- | --- | --- | --- | --- | --- | --- | --- |
| **Applicator Size (mm)** | **Monte Carlo** | **Roos** | **Markus** | **Advanced  Markus** | **PPC 05** | **PPC40** | **PPC NACP-02** | **Semiflex** | **Semiflex 3D** | **Pinpoint** | **microDiamond** | **Diode E** | **Diode SRS** | **Edge** |
| 30 | 1.630 | 1.588 | 1.667 | 1.662 | 1.599 | 1.566 | 1.336 | 1.699 | 1.704 | 1.645 | 1.642 | 1.650 | 1.061 | 1.650 |
| 40 | 1.590 | 1.603 | 1.629 | 1.609 | 1.576 | 1.597 | 1.322 | 1.675 | 1.655 | 1.654 | 1.607 | 1.589 | 1.060 | 1.609 |
| 50 | 1.520 | 1.562 | 1.564 | 1.547 | 1.543 | 1.565 | 1.301 | 1.611 | 1.590 | 1.558 | 1.533 | 1.539 | 1.060 | 1.547 |
| 60 | 1.400 | 1.427 | 1.426 | 1.423 | 1.403 | 1.444 | 1.172 | 1.462 | 1.440 | 1.417 | 1.404 | 1.406 | 1.050 | 1.416 |
| 70 | 1.280 | 1.304 | 1.301 | 1.302 | 1.291 | 1.314 | 1.105 | 1.337 | 1.307 | 1.316 | 1.282 | 1.282 | 1.041 | 1.295 |
| 80 | 1.170 | 1.173 | 1.177 | 1.184 | 1.173 | 1.194 | 1.042 | 1.205 | 1.182 | 1.212 | 1.175 | 1.178 | 1.028 | 1.187 |
| 100 | 1.000 | 1.000 | 1.000 | 1.000 | 1.000 | 1.000 | 1.000 | 1.000 | 1.000 | 1.000 | 1.000 | 1.000 | 1.000 | 1.000 |
| **Measured OF and OF_MC_ Δ% difference for 10 MeV** | | | | | | | | | | | | | | |
| **Applicator Size (mm)** | **Monte Carlo** | **Roos** | **Markus** | **Advanced Markus** | **PPC 05** | **PPC40** | **PPC NACP-02** | **Semiflex** | **Semiflex 3D** | **Pinpoint** | **microDiamond** | **Diode E** | **Diode SRS** | **Edge** |
| 30 | **0.0** | **-2.5** | **2.3** | **2.0** | **-1.9** | -4.0 | -18.0 | 4.2 | 4.6 | **0.9** | **0.7** | **1.2** | -34.9 | **1.2** |
| 40 | **0.0** | **0.8** | **2.4** | **1.2** | **-0.9** | **0.4** | -16.8 | 5.3 | 4.1 | 4.0 | **1.1** | **-0.1** | -33.3 | **1.2** |
| 50 | **0.0** | 2.8 | 2.9 | **1.8** | **1.5** | **2.9** | -14.4 | 6.0 | 4.6 | **2.5** | **0.9** | **1.3** | -30.3 | **1.8** |
| 60 | **0.0** | **1.9** | **1.9** | **1.6** | **0.2** | **3.1** | -16.3 | 4.4 | 2.8 | **1.2** | **0.3** | **0.4** | -25.0 | **1.1** |
| 70 | **0.0** | **1.8** | **1.6** | **1.7** | **0.8** | **2.7** | -13.7 | 4.5 | **2.1** | **2.8** | **0.2** | **0.1** | -18.7 | **1.2** |
| 80 | **0.0** | **0.3** | **0.6** | **1.2** | **0.3** | **2.1** | -11.0 | 3.0 | **1.0** | **3.6** | **0.4** | **0.7** | -12.1 | **1.5** |
| 100 | **0.0** | **0.0** | **0.0** | **0.0** | **0.0** | **0.0** | **0.0** | **0.0** | **0.0** | **0.0** | **0.0** | **0.0** | **0.0** | **0.0** |

*Table7. Measured and k_sat_ corrected OFs for flat applicators at 10 MeV of all detectors and OF_MC_ results. Δ% is the percentage difference of measured OFs to OF_MC_*.

| *k_sat_* for 10 MeV | | | | | | | | | |
| --- | --- | --- | --- | --- | --- | --- | --- | --- | --- |
| Applicator (mm) | Roos | Markus | Advanced Markus | Semiflex | Semiflex3D | Pinpoint | PPC 05 | PPC40 | PPC NACP-02 |
| **30** | 1.288 | 1.214 | 1.045 | 1.252 | 1.385 | 1.033 | 1.014 | 1.177 | 1.452 |
| **40** | 1.308 | 1.210 | 1.043 | 1.259 | 1.379 | 1.033 | 1.018 | 1.181 | 1.447 |
| **50** | 1.302 | 1.203 | 1.041 | 1.248 | 1.365 | 1.023 | 1.016 | 1.178 | 1.439 |
| **60** | 1.280 | 1.190 | 1.038 | 1.222 | 1.332 | 1.025 | 1.015 | 1.165 | 1.423 |
| **70** | 1.256 | 1.172 | 1.036 | 1.203 | 1.296 | 1.027 | 1.015 | 1.150 | 1.402 |
| **80** | 1.220 | 1.152 | 1.030 | 1.178 | 1.264 | 1.021 | 1.013 | 1.135 | 1.377 |
| **100** | 1.195 | 1.135 | 1.026 | 1.152 | 1.229 | 1.016 | 1.012 | 1.114 | 1.357 |

*Table8. k_sat_ of ion chambers with different applicator sizes for 10 MeV*

| **Measured OF and OF_MC_ for 12 MeV** | | | | | | | | | | | | | | |
| --- | --- | --- | --- | --- | --- | --- | --- | --- | --- | --- | --- | --- | --- | --- |
| **Applicator Size (mm)** | **Monte Carlo** | **Roos** | **Markus** | **Advanced  Markus** | **PPC 05** | **PPC40** | **PPC NACP-02** | **Semiflex** | **Semiflex 3D** | **Pinpoint** | **microDiamond** | **Diode E** | **Diode SRS** | **Edge** |
| 30 | 1.670 | 1.679 | 1.745 | 1.700 | 1.657 | 1.653 | 1.461 | 1.758 | 1.744 | 1.671 | 1.683 | 1.708 | 1.050 | 1.691 |
| 40 | 1.610 | 1.647 | 1.676 | 1.633 | 1.612 | 1.653 | 1.447 | 1.728 | 1.682 | 1.592 | 1.602 | 1.617 | 1.050 | 1.685 |
| 50 | 1.520 | 1.605 | 1.577 | 1.550 | 1.550 | 1.605 | 1.426 | 1.644 | 1.592 | 1.550 | 1.535 | 1.556 | 1.048 | 1.666 |
| 60 | 1.390 | 1.450 | 1.452 | 1.417 | 1.415 | 1.455 | 1.312 | 1.490 | 1.427 | 1.413 | 1.405 | 1.415 | 1.039 | 1.425 |
| 70 | 1.280 | 1.315 | 1.320 | 1.295 | 1.297 | 1.315 | 1.202 | 1.359 | 1.298 | 1.294 | 1.280 | 1.284 | 1.033 | 1.265 |
| 80 | 1.170 | 1.207 | 1.196 | 1.171 | 1.178 | 1.182 | 1.069 | 1.201 | 1.173 | 1.191 | 1.170 | 1.171 | 1.018 | 1.177 |
| 100 | 1.000 | 1.000 | 1.000 | 1.000 | 1.000 | 1.000 | 1.000 | 1.000 | 1.000 | 1.000 | 1.000 | 1.000 | 1.000 | 1.000 |
| **Measured OF and OF_MC_ Δ% difference for 12 MeV** | | | | | | | | | | | | | | |
| **Applicator Size (mm)** | **Monte Carlo** | **Roos** | **Markus** | **Advanced Markus** | **PPC 05** | **PPC40** | **PPC NACP-02** | **Semiflex** | **Semiflex 3D** | **Pinpoint** | **microDiamond** | **Diode E** | **Diode SRS** | **Edge** |
| 30 | **0.0** | **0.5** | 4.5 | **1.8** | **-0.8** | **-1.0** | -12.5 | 5.3 | 4.4 | **0.1** | **0.8** | **2.3** | -37.1 | **1.3** |
| 40 | **0.0** | 2.3 | 4.1 | **1.4** | **0.1** | 2.7 | -10.1 | 7.3 | 4.5 | **-1.1** | **-0.5** | **0.4** | -34.8 | 4.7 |
| 50 | **0.0** | 5.6 | 3.7 | **2.0** | **2.0** | 5.6 | -6.2 | 8.1 | 4.8 | **2.0** | **1.0** | **2.4** | -31.0 | 9.6 |
| 60 | **0.0** | 4.3 | 4.4 | **1.9** | **1.8** | 4.7 | -5.6 | 7.2 | 2.7 | **1.6** | **1.1** | **1.8** | -25.3 | 2.5 |
| 70 | **0.0** | 2.7 | 3.1 | **1.2** | **1.3** | 2.7 | -6.1 | 6.2 | **1.4** | **1.1** | **0.0** | **0.3** | -19.3 | **-1.1** |
| 80 | **0.0** | 3.2 | **2.3** | **0.1** | **0.7** | **1.0** | -8.7 | 2.6 | **0.3** | **1.8** | **0.0** | **0.1** | -13.0 | **0.6** |
| 100 | **0.0** | **0.0** | **0.0** | **0.0** | **0.0** | **0.0** | **0.0** | **0.0** | **0.0** | **0.0** | **0.0** | **0.0** | **0.0** | **0.0** |

*Table9. Measured and k_sat_ corrected OFs for flat applicators at 12 MeV with all detectors and OF_MC_ results. Δ% is the percentage difference of measured OFs to OF_MC_.*

| *k_sat_* for 12 MeV | | | | | | | | | |
| --- | --- | --- | --- | --- | --- | --- | --- | --- | --- |
| Applicator (mm) | Roos | Markus | Advanced Markus | Semiflex | Semiflex3D | Pinpoint | PPC 05 | PPC40 | PPC NACP-02 |
| **30** | 1.467 | 1.303 | 1.066 | 1.392 | 1.594 | 1.052 | 1.019 | 1.293 | 1.694 |
| **40** | 1.474 | 1.295 | 1.068 | 1.406 | 1.588 | 1.050 | 1.018 | 1.290 | 1.676 |
| **50** | 1.477 | 1.284 | 1.062 | 1.388 | 1.552 | 1.050 | 1.018 | 1.289 | 1.643 |
| **60** | 1.435 | 1.260 | 1.056 | 1.355 | 1.500 | 1.047 | 1.015 | 1.260 | 1.615 |
| **70** | 1.396 | 1.246 | 1.051 | 1.325 | 1.451 | 1.044 | 1.013 | 1.230 | 1.567 |
| **80** | 1.346 | 1.223 | 1.041 | 1.275 | 1.410 | 1.040 | 1.010 | 1.200 | 1.510 |
| **100** | 1.299 | 1.198 | 1.030 | 1.233 | 1.363 | 1.037 | 1.007 | 1.180 | 1.468 |

*Table10. k_sat_ of ion chambers with different applicator sizes for 12 MeV*
